# 硼替佐米时代IgA型多发性骨髓瘤的临床特征与生存结局：一项单中心回顾性队列研究

**DOI:** 10.3760/cma.j.cn121090-20240924-00367

**Published:** 2025-08

**Authors:** 帆 高, 环 王, 玉兰 周, 诗轩 王, 敏 喻, 菲 李

**Affiliations:** 南昌大学第一附属医院血液科，江西省血液病临床医学研究中心，江西省血液系统疾病重点实验室，南昌 330006 Department of Hematology, The First Affiliated Hospital of Nanchang University, Institute of Hematology, Academy of Clinical Medicine of Jiangxi Province, Jiangxi Provincial Key Laboratory of Hematological Diseases, Nanchang 330006, China

**Keywords:** 多发性骨髓瘤, IgA型, 临床特征, 疗效, 预后分析, Multiple myeloma, IgA type, Clinical features, Efficacy, Prognostic analysis

## Abstract

**目的:**

分析初治IgA型多发性骨髓瘤（MM）患者的临床特征、治疗反应及预后，探索硼替佐米治疗时代IgA型是否依然为预后不良因素。

**方法:**

回顾性纳入2014年3月至2021年12月南昌大学第一附属医院血液科收治的155例IgA型和420例非IgA型初治MM患者，比较患者的临床特征及预后，评估接受不同治疗方案IgA型与非IgA型患者无进展生存（PFS）与总生存（OS）是否存在差异。

**结果:**

与非IgA型患者相比，IgA型患者呈现更具侵袭性的临床特征，包括HGB<85 g/L（61.3％对51.4％，*P*＝0.035），髓外病变（EMs）（20.0％对11.4％，*P*＝0.008）和gain/amp（1q21）（48.6％对36.7％，*P*＝0.032）的患者比例更高。疗效分析显示，IgA型患者的总反应率（ORR）低于非IgA型患者（83.2％对92.4％，*P*＝0.001）。在使用硼替佐米为基础治疗的IgA型与非IgA型患者中，ORR分别为91.2％和94.8％，差异无统计学意义（*P*＝0.146）。生存分析显示，IgA型患者中位PFS和OS期分别为23.5（95％ *CI*：17.4～29.5）个月和48.8（95％ *CI*：30.1～67.5）个月，显著短于非IgA型MM患者的40.7（95％ *CI*：33.8～47.6）个月和未达到（*P*<0.001、*P*＝0.002）。在硼替佐米未序贯自体造血干细胞移植（auto-HSCT）组，相较于非IgA型MM患者，IgA型患者PFS期［25.4（95％ *CI*：18.7～32.1）个月对41.0（95％ *CI*：33.7～48.3）个月，*P*＝0.001］和OS期［53.5（95％ *CI*：35.4～71.6）个月对未达到，*P*＝0.011］更短。然而，采取硼替佐米为基础的诱导化疗序贯auto-HSCT，IgA型MM患者的1年、3年和5年OS率分别为96％、81％和81％，而非IgA型MM患者则分别为93％、89％和79％（*P*＝0.758）。

**结论:**

在硼替佐米时代，IgA型MM因其固有的高危生物学特征，总体预后仍劣于非IgA型。尽管含硼替佐米的方案可有效提升治疗反应，但未能完全弥合生存差距。硼替佐米序贯auto-HSCT可能是克服IgA型预后不良，使其获得与非IgA型患者相当长期生存的关键策略。

## Abstract (en)

**Objective:**

To analyze the clinical characteristics, treatment response, and prognosis of patients newly diagnosed with immunoglobulin A multiple myeloma（IgA MM）, and to ascertain whether the IgA isotype remains a poor prognostic factor in the bortezomib era.

**Methods:**

This study retrospectively enrolled 155 patients newly diagnosed with IgA MM and 420 with non-IgA MM admitted to the Department of Hematology, the First Affiliated Hospital of Nanchang University from March 2014 to December 2021. We compared the two groups in terms of their clinical characteristics, prognoses, and progression-free survival（PFS）and overall survival（OS）following different treatment regimens.

**Results:**

Compared with the non-IgA group, the IgA group presented with more aggressive clinical features, including a higher proportion of patients with hemoglobin<85 g/L（61.3％ *vs* 51.4％, *P*＝0.035）, extramedullary manifestations（20.0％ *vs* 11.4％, *P*＝0.008）, and gain/amp（1q21）（48.6％ *vs* 36.7％, *P*＝0.032）. Efficacy analysis revealed a lower overall response rate（ORR）in the IgA group than in the non-IgA group（83.2％ *vs* 92.4％, *P*＝0.001）. Among patients treated with bortezomib-based regimens, the ORR was 91.2％ in the IgA group and 94.8％ in the non-IgA group, but the difference was nonsignificant（*P*＝0.146）. Survival analysis showed that the median PFS and OS were significantly shorter in the IgA group compared with the non-IgA group［23.5（95％ *CI*: 17.4–29.5）months and 48.8（95％ *CI*: 30.1–67.5）months *vs* 40.7（95％ *CI*: 33.8–47.6）months and not reached, respectively; *P*<0.001 and *P*＝0.002］. In the subgroup of patients who received bortezomib-based therapy without subsequent autologous hematopoietic stem cell transplantation（auto-HSCT）, the PFS and OS were significantly shorter in the IgA group compared with the non-IgA group［25.4（95％ *CI*: 18.7–32.1）months and 53.5（95％ *CI*: 35.4–71.6）months *vs* 41.0（95％ *CI*: 33.7–48.3）months and not reached; *P*＝0.001 and *P*＝0.011］. In patients who underwent bortezomib-based induction therapy followed by auto-HSCT, the 1-, 3-, and 5-year OS rates for the IgA group were 96％, 81％, and 81％, respectively, compared with 93％, 89％, and 79％ for the non-IgA group, but the difference was nonsignificant（*P*＝0.758）.

**Conclusion:**

In the bortezomib era, IgA MM is still associated with a poorer overall prognosis than non-IgA MM, likely due to its inherent high-risk biological characteristics. Although bortezomib-based regimens effectively improve the treatment response, they fail to completely bridge the survival gap between the two disease isotypes. Therefore, bortezomib-based therapy followed by auto-HSCT may be a key strategy to overcome the poor prognosis of IgA MM, potentially enabling these patients to achieve long-term survival comparable to that of their non-IgA counterparts.

多发性骨髓瘤（MM）是一种具有高度临床及生物学异质性的恶性浆细胞疾病，根据分泌的免疫球蛋白重链和（或）轻链类型被归为不同亚型。其中，IgA型是仅次于IgG型的第二常见亚型，占所有MM的16.7％～33.3％，表现出更高的生物学侵袭性[1]–[2]。根据既往文献报道，IgA型MM患者总生存（OS）期显著短于IgG型，提示免疫亚型是重要的预后影响因素[3]。近年来，随着MM生物学研究的重大突破和个体化治疗策略的显著进展，MM患者的整体预后已得到明显改善[4]–[8]。然而，在硼替佐米为基础的治疗时代，IgA型相较于其他亚型的生存劣势是否依然存在，国内尚缺乏基于大样本、长期随访的临床研究证据。为此，本研究基于中国单中心较大样本队列，回顾性分析初治IgA型MM患者的临床资料，旨在评估其发病特征、临床特点及生存结局，探索硼替佐米治疗时代IgA型是否依然为预后不良因素，以期为IgA型MM的精准诊疗提供理论依据和实践指导。

## 病例与方法

一、病例资料

本研究为回顾性队列研究，纳入2014年3月至2021年12月在南昌大学第一附属医院接受治疗的155例IgA型MM患者和420例非IgA型（IgG型301例、轻链型119例）MM患者。纳入标准：①所有患者均符合国际骨髓瘤工作组（IMWG）诊断标准；②2014年3月至2021年12月期间在南昌大学第一附属医院接受系统治疗并进行疗效评估患者或通过电话随访获得上述信息者；③年龄≥18岁。排除标准：①合并其他恶性肿瘤；②严重的心肺脑疾病，器官移植术后；③复发/难治性MM。根据Durie-Salmon分期（D-S分期）和国际分期系统（ISS分期）进行分期[9]–[10]。收集患者的临床资料，包括年龄、性别、血常规、血生化、血清免疫固定电泳、影像学检查、骨髓浆细胞比例、骨髓免疫分型和荧光原位杂交（FISH）结果。使用血清免疫固定电泳对血清中的各种蛋白成分进行分离，用于区分M蛋白的类型。本研究将肌酐≥177 µmol/L定义为肾功能异常。

二、治疗方案

诱导治疗方案分为非硼替佐米为基础的诱导方案（非硼替佐米组）和硼替佐米为基础的诱导方案（硼替佐米组）。非硼替佐米组包括VAD（阿霉素、长春新碱和地塞米松）、RD（来那度胺和地塞米松）、以沙利度胺为基础方案（TD：沙利度胺和地塞米松；TAD：沙利度胺、阿霉素和地塞米松；TCD：沙利度胺、环磷酰胺和地塞米松；MPT：美法仑、泼尼松和沙利度胺）。硼替佐米组包括BD（硼替佐米和地塞米松）、PAD（硼替佐米、阿霉素和地塞米松）、BCD（硼替佐米、环磷酰胺和地塞米松）、VTD（硼替佐米、沙利度胺和地塞米松）和VRD（硼替佐米、来那度胺和地塞米松）。部分适合自体造血干细胞移植（auto-HSCT）的患者接受至少4个周期治疗后，疗效达PR及以上序贯auto-HSCT，其余患者继续原方案巩固治疗，维持治疗药物包括硼替佐米/伊沙佐米/来那度胺/沙利度胺。

三、疗效评估

疗效评估采用IMWG标准[11]，分为严格意义的完全缓解（sCR），完全缓解（CR）、非常好的部分缓解（VGPR）、部分缓解（PR）、疾病稳定（SD）、疾病进展（PD）。总反应率（ORR）为sCR、CR、VGPR、PR率之和。

四、随访

通过门诊、住院复查及电话对患者进行随访，主要随访指标包括复发/进展、是否生存、治疗情况（是否用药、具体方案），随访频率为6个月直至患者死亡。随访截至2024年5月31日，中位随访时间为46.3（0.5～126.4）个月，在截止日期前失访的患者，其生存数据在最后一次确认存活的时间点进行截尾。无进展生存（PFS）期定义为从疾病诊断至任何原因导致疾病进展、死亡的时间或随访终点。OS期定义为从疾病诊断至因任何原因死亡或随访终止的时间。

五、统计学处理

采用SPSS 26.0软件进行统计学分析。不符合正态分布的连续变量以中位数（范围）表示，分类变量以例数（构成比）表示。分类变量组间比较采用卡方检验或Fisher精确概率法。采用Kaplan-Meier绘制生存曲线，单因素组间比较采用Log-rank检验；我们进一步进行了亚组分析，以探究各治疗方案对IgA型和非IgA型患者生存差异的影响。*P*<0.05为差异有统计学意义。

## 结果

一、临床特征

IgA型MM患者的中位年龄为60（32～81）岁。与非IgA型MM患者相比，IgA型MM患者HGB<85 g/L（61.3％对51.4％，*P*＝0.035）和髓外病变（EMs）（20.0％对11.4％，*P*＝0.008）比例更高，更易合并gain/amp（1q21）（48.6％对36.7％，*P*＝0.032），且合并肾功能异常比例更低（15.5％对23.3％，*P*＝0.041）。两组患者其他临床特征见表1。

**表1:** IgA型和非IgA型多发性骨髓瘤患者的基线特征［有效例数/总例数（％）］

临床特征	IgA型（155例）	非IgA型（420例）	*P*值
年龄			0.124
<65岁	106/155（68.4）	258/420（61.4）	
≥65岁	49/155（31.6）	162/420（38.6）	
性别			0.788
女	68/155（43.8）	179/420（42.6）	
男	87/155（56.2）	241/420（57.4）	
HGB			0.035
<85 g/L	95/155（61.3）	216/420（51.4）	
≥85 g/L	60/155（38.7）	204/420（48.6）	
LDH			0.157
<220 U/L	104/155（67.1）	307/420（73.1）	
≥220 U/L	51/155（32.9）	113/420（26.9）	
肌酐			0.041
<177 µ mol/L	131/155（84.5）	322/420（76.7）	
≥177 µ mol/L	24/155（15.5）	98/420（23.3）	
白蛋白			0.107
<30 g/L	59/155（38.1）	130/420（31.0）	
≥30 g/L	96/155（61.9）	290/420（69.0）	
骨髓浆细胞比例			0.459
<50％	130/155（83.9）	341/420（81.2）	
≥50％	25/155（16.1）	79/420（18.8）	
溶骨性损害			0.896
是	121/155（78.1）	330/420（78.6）	
否	34/155（21.9）	90/420（21.4）	
髓外病变			0.008
是	31/155（20.0）	48/420（11.4）	
否	124/155（80.0）	372/420（88.6）	
轻链类型			0.541
κ型	86/155（55.5）	221/420（52.6）	
λ型	69/155（44.5）	199/420（47.4）	
D-S分期			0.800
Ⅰ+Ⅱ期	17/155（11.0）	43/420（10.2）	
Ⅲ期	138/155（89.0）	377/420（89.8）	
ISS分期			0.563
Ⅰ+Ⅱ期	87/155（56.1）	247/420（58.8）	
Ⅲ期	68/155（43.9）	173/420（41.2）	
诱导方案			
非硼替佐米组			0.751
传统化疗	7/30（23.3）	11/54（20.4）	
IMiDs±传统化疗	23/30（76.7）	43/54（79.6）	
硼替佐米组			0.709
硼替佐米±传统化疗	96/125（76.8）	275/366（75.1）	
硼替佐米+IMiDs±传统化疗	29/125（23.2）	91/366（24.9）	
自体造血干细胞移植			0.581
是	25/155（16.1）	60/420（14.2）	
否	130/155（83.9）	360/420（85.8）	
细胞遗传学异常	69/109（63.3）	158/267（59.2）	0.458
gain/amp（1q21）	53/109（48.6）	98/267（36.7）	0.032
IGH重排	28/109（25.7）	95/267（35.6）	0.064
del（13q）	30/109（27.5）	86/267（32.2）	0.372
del（17p）	10/109（9.2）	27/267（10.1）	0.782

**注** LDH：乳酸脱氢酶；D-S分期：Durie-Salmon分期；ISS分期：国际分期系统；IMiDs：免疫调节剂；传统化疗包括阿霉素、长春新碱和地塞米松；IMiDs±传统化疗包括来那度胺和地塞米松，沙利度胺和地塞米松，沙利度胺、阿霉素和地塞米松，沙利度胺、环磷酰胺和地塞米松，美法仑、泼尼松和沙利度胺；硼替佐米±传统化疗包括硼替佐米和地塞米松，硼替佐米、阿霉素和地塞米松，硼替佐米、环磷酰胺和地塞米松；硼替佐米+IMiDs包括硼替佐米、沙利度胺和地塞米松，硼替佐米、来那度胺和地塞米松

二、疗效评估

在155例IgA型MM患者中，125例患者接受以硼替佐米为基础的治疗，30例患者接受以非硼替佐米为基础的治疗；在420例非IgA型MM患者中，366例患者接受以硼替佐米为基础的治疗方案，54例患者接受以非硼替佐米为基础的治疗。所有接受治疗的患者中评估最佳疗效，IgA型MM患者ORR低于非IgA型MM患者（83.2％对92.4％，*P*＝0.001）。硼替佐米组中，IgA型与非IgA型患者ORR差异无统计学意义（91.2％对94.8％，*P*＝0.146）；非硼替佐米组中，IgA型MM患者ORR低于非IgA型MM患者（50.0％对75.9％，*P*＝0.023）（表2）。提示IgA型MM患者可通过硼替佐米为基础的治疗提升疗效，并获得与非IgA型MM患者一致的疗效。

**表2:** 是否接受硼替佐米为基础治疗IgA型与非IgA型多发性骨髓瘤患者的总体疗效比较［有效例数/总例数（％）］

组别	IgA型	非IgA型	*P*值
总体	129/155（83.2）	388/420（92.4）	0.001
硼替佐米组	114/125（91.2）	347/366（94.8）	0.146
非硼替佐米组	15/30（50.0）	41/54（75.9）	0.023

**注** 非硼替佐米组包括阿霉素、长春新碱和地塞米松，来那度胺和地塞米松，沙利度胺和地塞米松，沙利度胺、阿霉素和地塞米松，沙利度胺、环磷酰胺和地塞米松，美法仑、泼尼松和沙利度胺；硼替佐米组包括硼替佐米和地塞米松，硼替佐米、阿霉素和地塞米松，硼替佐米、环磷酰胺和地塞米松，硼替佐米、沙利度胺和地塞米松，硼替佐米、来那度胺和地塞米松

三、生存分析

IgA型MM患者中位PFS期和OS期分别为23.5（95％ *CI*：17.4～29.5）个月和48.8（95％ *CI*：30.1～67.5）个月。非IgA型MM患者中位PFS期和OS期分别为40.7（95％ *CI*：33.8～47.6）个月和未达到。两组PFS和OS差异有统计学意义（*P*<0.001、*P*＝0.002）（图1A、B），IgA型较非IgA型MM患者PFS和OS更差。

**图1:**
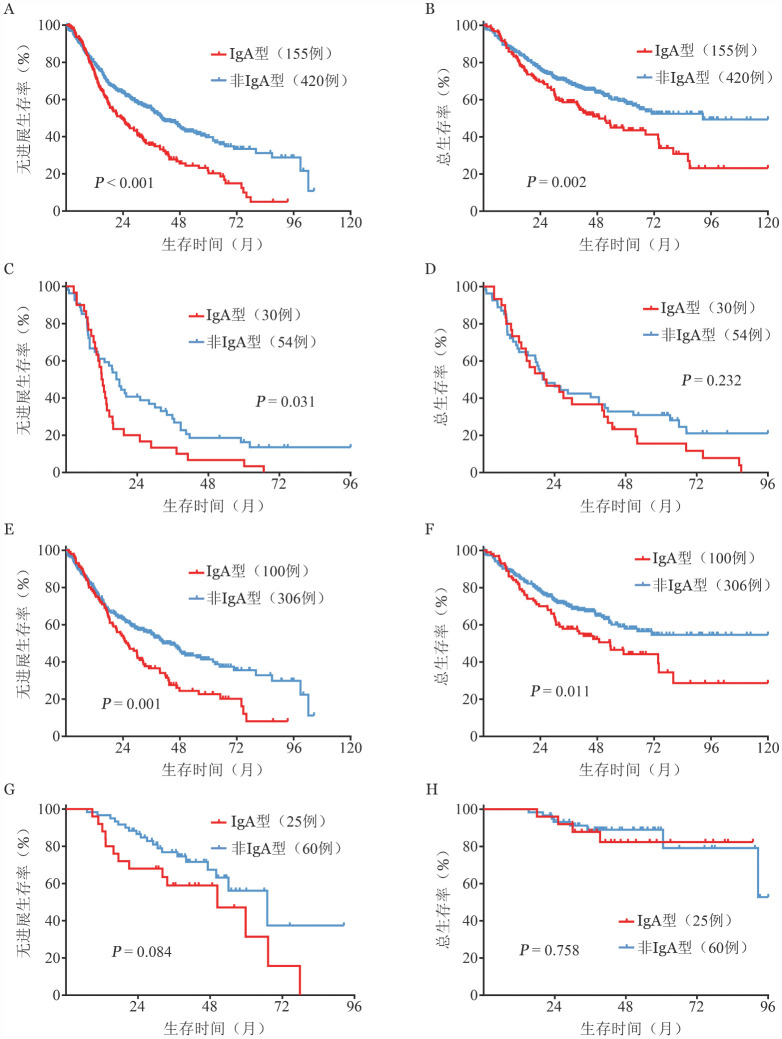
IgA和非IgA型多发性骨髓瘤患者的无进展生存和总生存曲线 **A、B** 总体患者；**C、D** 非硼替佐米亚组；**E、F** 硼替佐米未序贯auto-HSCT亚组；**G、H** 硼替佐米序贯auto-HSCT亚组 **注** 非硼替佐米组包括阿霉素、长春新碱和地塞米松，来那度胺和地塞米松，沙利度胺和地塞米松，沙利度胺、阿霉素和地塞米松，沙利度胺、环磷酰胺和地塞米松，美法仑、泼尼松和沙利度胺；硼替佐米组包括硼替佐米和地塞米松，硼替佐米、阿霉素和地塞米松，硼替佐米、环磷酰胺和地塞米松，硼替佐米、沙利度胺和地塞米松，硼替佐米、来那度胺和地塞米松；auto-HSCT：自体造血干细胞移植

在非硼替佐米亚组中，IgA型（30例）较非IgA型（54例）MM患者PFS期短［12.0（95％ *CI*：10.0～14.0）个月对17.0（95％ *CI*：13.3～20.7）个月，*P*＝0.031］，但OS期差异无统计学意义［20.4（95％ *CI*：7.0～33.8）个月对20.0（95％ *CI*：11.2～28.8）个月，*P*＝0.232］（图1C、D）。

在硼替佐米未序贯auto-HSCT亚组中，IgA型（100例）较非IgA型（306例）MM患者PFS期［25.4（95％ *CI*：18.7～32.1）个月对41.0（95％ *CI*：33.7～48.3）个月，*P*＝0.001］和OS期［53.5（95％ *CI*：35.4～71.6）个月对未达到，*P*＝0.011］更短（图1E、F）。

为了探讨以硼替佐米为基础的诱导化疗序贯auto-HSCT对IgA型MM患者的影响，我们分析了25例IgA型和60例非IgA型MM患者，结果显示IgA型MM患者中位PFS期和OS期分别为50.4（95％ *CI*：25.7～75.1）个月和未达到。非IgA型MM患者中位PFS期和OS期分别为67.0（95％ *CI*：44.6～89.4）个月和未达到。两组之间的PFS和OS差异无统计学意义（*P*＝0.084和*P*＝0.758）。IgA型MM患者的1年、3年和5年OS率分别为96％、81％和81％，而非IgA型MM患者则分别为93％、89％和79％。提示以硼替佐米为基础的诱导化疗序贯auto-HSCT或可使IgA型MM患者达到与非IgA型MM患者类似的生存（图1G、H）。

## 讨论

近些年MM的治疗取得了显著进展，生存得到明显改善[8]–[9]。危险分层体系不断完善，常常基于分子遗传学和临床指标采用D-S分期、ISS分期和修订后的ISS分期来评估MM患者的预后[10]–[13]。多项研究提示，不同免疫学分型的MM具有异质性的临床特征及预后结局，其中IgA型在硼替佐米治疗时代仍显示出相对不良的生存预后[14]–[15]。然而，既往研究多将IgA型纳入整体MM队列进行统一分析，受限于该亚型病例数较少，其独特的临床生物学行为及生存模式尚未得到深入阐明。

本研究基于南昌大学第一附属医院575例初治MM患者的临床资料，其中IgA型患者达155例（27.0％），为深入解析该亚型特征提供了较大样本量的依据。本研究证实，IgA型MM患者展现出区别于其他亚型的独特临床与生物学特征。本研究中IgA型MM患者的中位发病年龄为60岁，这与国内多项报道一致，显著低于欧美国家报告的65～70岁[15]–[16]。此外，贫血（HGB<110 g/L）是MM各亚型的常见临床表现，但在IgA型患者中程度更为严重。本研究队列数据显示，IgA型患者进展至中重度贫血（HGB<85 g/L）的比例显著增高，其机制主要与该亚型固有的侵袭性生物学行为相关。尽管贫血未被确立为独立预后因子，但其诱发的慢性组织缺氧可导致多器官功能进行性损伤（尤其心肾系统），显著降低患者生活质量并削弱治疗耐受性[17]。值得注意的是，在本研究队列中，IgA型患者肾功能损害的发生率为15.5％，低于非IgA型患者的23.3％。深入分析发现，这一差异主要源于非IgA组中轻链型MM患者较高的肾功能不全发生率（39.5％），该比例甚至高于法国一项大型研究报道的32.5％[18]。提示在评估MM肾脏并发症发生率及风险因素时，若未充分考虑不同免疫分型（特别是轻链型占比）的构成差异，研究结论可能受到干扰。因此，未来相关研究应依据M蛋白类型构建分层分析模型，以获得更精准的结论。

治疗反应方面，Wang等[15]研究发现，相较于非硼替佐米治疗方案，以硼替佐米为基础的治疗可将IgA型MM患者的ORR从55.0％提高至95.5％。本研究也证实，硼替佐米为基础的治疗可将IgA型患者的ORR从50.0％提升至91.2％，但尽管如此，IgA型患者的中位OS期仍然短于非IgA型患者，这也与Bal等[14]的研究结果一致，即使在蛋白酶体抑制剂和免疫调节剂等新型药物的背景下，IgA型MM的OS仍然较差。这一现象的潜在原因可能与IgA型特征性的分子遗传学病变相关，本研究发现细胞遗传学异常在IgA型MM中的特征性分布。IgA型患者细胞遗传学异常检出率达63.3％，其中gain/amp（1q21）（占比48.6％）最为常见，这与国内多数研究结果高度吻合[14],[19]。值得注意的是，欧美研究报道IgA型患者中t（4;14）发生率较高，但本中心因伴IGH重排病例的FISH检测率不足，未能深入分析该差异[20]–[21]。现有证据表明，gain/amp（1q21）是继发性EMs的独立预后因素，且该基因异常通过激活MAPK/ERK信号通路，上调CKSB1和MCL-1的表达，从而增强疾病的侵袭性[22]。但是，EMs与特定遗传学异常的精确互作机制仍需深入探索。本队列中IgA型患者EMs发生率为20.0％，虽低于Wang等[15]报道的31.9％，但显著高于非IgA型患者的11.4％（*P*<0.05）。需指出的是，本中心PET/CT使用率相对较低，可能影响髓外包块检出的敏感性。更深层的免疫学差异亦不容忽视，Gkoliou等[23]通过免疫球蛋白基因库分析发现，IgA型与IgG型MM呈现不同的体细胞高频突变（SHM）模式。研究者推测，差异性的抗原暴露轨迹与抗体亲和力成熟过程可能共同塑造疾病演进路径。值得注意的是，gain/amp（1q21）、t（4;14）等高危遗传事件可能通过干扰免疫球蛋白基因表达与SHM过程，共同加剧疾病进展。

本研究发现，接受硼替佐米诱导后序贯auto-HSCT的IgA型患者，其OS与非IgA型患者相当，提示auto-HSCT可能在一定程度上克服IgA型患者的不良预后。然而，本研究仅有25例IgA型患者接受了auto-HSCT，现有样本量的局限性使得我们难以对EMs或gain/amp（1q21）等关键预后因素进行亚组分析。本研究还存在一定的局限性，治疗方案选择受患者体能状态、年龄、合并症（尤其心肾功能）及社会经济因素（药物可及性与移植意愿）等多重因素影响。例如，接受auto-HSCT的IgA型患者本身代表了体能状态更好、并发症更少、经济条件允许的特定亚群，其获得的良好生存结局可能无法完全外推至整体IgA型人群，特别是体能状态较差或经济受限的患者。本中心收治的患者群体不可避免地带有地域性和特定的转诊模式，其疾病特征谱可能与全国整体IgA型MM人群存在差异。同时，新型免疫靶向药物如CD38单抗（如达雷妥尤单抗）、双特异性抗体和CAR-T细胞治疗等新型疗法已经显示出突破性疗效[24]–[26]。有研究表明达雷妥尤单抗在新诊断患者中的广泛应用，gain/amp（1q21）带来的不良预后影响正逐步减弱[27]–[28]。因此，未来应扩大样本量，评估以CD38单抗为基础的治疗是否能够改善IgA型MM患者的预后，并通过精细化的MM分期为患者提供个性化的治疗方案。

## References

[1] Siegel RL, Miller KD, Wagle NS (2023). Cancer statistics, 2023[J]. CA Cancer J Clin.

[2] Malard F, Neri P, Bahlis NJ (2024). Multiple myeloma. Nat Rev Dis Primers.

[3] Zhang L, Qi JY, Qi PJ (2010). Comparison among immunologically different subtypes of 595 untreated multiple myeloma patients in northern China[J]. Clin Lymphoma Myeloma Leuk.

[4] 解 琳娜, 王 鑫, 贺 强 (2024). 国产硼替佐米联合来那度胺、地塞米松治疗初治多发性骨髓瘤的多中心、前瞻性、Ⅱ期、单臂研究[J]. 中华血液学杂志.

[5] 中国医师协会血液科医师分会, 中华医学会血液学分会 (2024). 中国多发性骨髓瘤诊治指南(2024年修订)[J]. 中华内科杂志.

[6] Kocoglu MH, Badros AZ (2020). Newly diagnosed multiple myeloma: current treatment strategies, emerging therapeutic approaches and beyond[J]. Expert Rev Hematol.

[7] Padala SA, Barsouk A, Barsouk A (2021). Epidemiology, Staging, and Management of Multiple Myeloma[J]. Med Sci (Basel).

[8] Mian H, Reece D, Masih-Khan E (2022). Survival and Outcomes of Newly Diagnosed Multiple Myeloma Patients Stratified by Transplant Status 2007-2018: Retrospective Analysis from the Canadian Myeloma Research Group Database[J]. Clin Lymphoma Myeloma Leuk.

[9] Das S, Juliana N, Yazit N (2022). Multiple Myeloma: Challenges Encountered and Future Options for Better Treatment[J]. Int J Mol Sci.

[10] Greipp PR, San Miguel J, Durie BG (2005). International staging system for multiple myeloma[J]. J Clin Oncol.

[11] Durie BG, Salmon SE (1975). A clinical staging system for multiple myeloma. Correlation of measured myeloma cell mass with presenting clinical features, response to treatment, and survival[J]. Cancer.

[12] Palumbo A, Avet-Loiseau H, Oliva S (2015). Revised International Staging System for Multiple Myeloma: A Report From International Myeloma Working Group[J]. J Clin Oncol.

[13] D'Agostino M, Cairns DA, Lahuerta JJ (2022). Second Revision of the International Staging System (R2-ISS) for Overall Survival in Multiple Myeloma: A European Myeloma Network (EMN) Report Within the HARMONY Project[J]. J Clin Oncol.

[14] Bal S, Giri S, Godby KN (2022). Revisiting the impact of immunoglobulin isotypes in multiple myeloma[J]. Ann Hematol.

[15] Wang L, Jin FY, Li Y (2018). IgA Type Multiple Myeloma, Clinical Features, and Prognosis[J]. Chin Med J (Engl).

[16] Cowan AJ, Green DJ, Kwok M (2022). Diagnosis and Management of Multiple Myeloma: A Review[J]. JAMA.

[17] Huang H, Yu PY, Wei C (2023). Regulatory Effect and Mechanism of Erythroblastic Island Macrophages on Anemia in Patients with Newly Diagnosed Multiple Myeloma[J]. J Inflamm Res.

[18] Courant M, Orazio S, Monnereau A (2021). Incidence, prognostic impact and clinical outcomes of renal impairment in patients with multiple myeloma: a population-based registry[J]. Nephrol Dial Transplant.

[19] Chen H, Zhou N, Shi H (2023). Presentation and outcomes of patients with multiple myeloma harboring gain or amplification of 1q21 and receiving novel agent therapies: results from a single-center study[J]. Hematology.

[20] Agbuduwe C, Iqbal G, Cairns D (2022). Clinical characteristics and outcomes of IgD myeloma: experience across UK national trials[J]. Blood Adv.

[21] Karlin L, Soulier J, Chandesris O (2011). Clinical and biological features of t(4;14) multiple myeloma: a prospective study[J]. Leuk Lymphoma.

[22] Zanwar S, Ho M, Lin Y (2023). Natural history, predictors of development of extramedullary disease, and treatment outcomes for patients with extramedullary multiple myeloma[J]. Am J Hematol.

[23] Gkoliou G, Agathangelidis A, Karakatsoulis G (2023). Differences in the immunoglobulin gene repertoires of IgG versus IgA multiple myeloma allude to distinct immunopathogenetic trajectories[J]. Front Oncol.

[24] Sun F, Cheng Y, Wanchai V (2024). Bispecific BCMA/CD24 CAR-T cells control multiple myeloma growth[J]. Nat Commun.

[25] van de Donk N, Zweegman S (2023). T-cell-engaging bispecific antibodies in cancer[J]. Lancet.

[26] Chen W, Cai Z, Chim JC (2025). Consensus Guidelines and Recommendations for The CD38 Monoclonal Antibody-based Quadruplet Therapy and Management in Clinical Practice for Newly Diagnosed Multiple Myeloma: From the Pan-Pacific Multiple Myeloma Working Group[J]. Clin Hematol Int.

[27] Callander NS, Silbermann R, Kaufman JL (2024). Daratumumab-based quadruplet therapy for transplant-eligible newly diagnosed multiple myeloma with high cytogenetic risk[J]. Blood Cancer J.

[28] Chari A, Kaufman JL, Laubach J (2024). Daratumumab in transplant-eligible patients with newly diagnosed multiple myeloma: final analysis of clinically relevant subgroups in GRIFFIN[J]. Blood Cancer J.

